# Dexamethasone Resisted Podocyte Injury via Stabilizing TRPC6 Expression and Distribution

**DOI:** 10.1155/2012/652059

**Published:** 2012-04-01

**Authors:** Shengyou Yu, L. Yu

**Affiliations:** Guangzhou Medical College, Guangzhou First Municipal People's Hospital, Guangdong Province, Guangzhou 510180, China

## Abstract

TRPC6, a member of the canonical transient receptor potential channel (TRPC) subfamily, is an important cation selective ion channel on podocytes. Podocytes are highly differentiated cells located on the visceral face of glomerular basement membrane and featured by numerous foot processes, on which nephrin, podocin, and TRPC6 locate. Podocytes and the slit diaphragm (SD) between adjacent foot processes form a selective filtration barrier impermeable to proteins. TRPC6 is very critical for normal podocyte function. To investigate the function of TRPC6 in podocytes and its relation to proteinuria in kidney diseases, we over-expressed TRPC6 in podocytes by puromycin aminonucleoside (PAN) and observed the changes of foot processes, TRPC6 protein distribution, and mRNA expression. Accordingly, in this study, we further investigated the role of specific signaling mechanisms underlying the prosurvival effects of dexamethasone (DEX) on podocyte repair. Our results showed that podocytes processes of overexpressing TRPC6 were reduced remarkably. These changes could be rescued by DEX via blocking TRPC6 channel. Additionally, our results also showed an improvement in TRPC6 arrangement in the cells and decrease of mRNA expression and protein distribution. From these results, we therefore proposed that overexpression of TRPC6 in podocytes may be one of the fundamental changes relating to the dysfunction of the SD and proteinuria. DEX may be maintained the structure and function integrity of SD by blocking TRPC6 signal pathway and played an important role in mechanisms of anti-proteinuria.

## 1. Introduction

TRPC6 is determined in recent years which is closely related with proteinuria and positioned in the structure protein molecules of SD. Proteinuria is a common feature of kidney dysfunction of glomerular origin and is itself a risk factor for renal disease [1]. There is a growing body of experimental and clinical literature showing that podocyte number is a critical determinant for the development of proteinuria and glomerulosclerosis [2]. PAN is used to induce a well-established cell model for podocytes injury. DEX is widely used for the treatment of a variety of glomerular diseases characterized by podocyte injury and proteinuria, including membranous nephropathy, minimal change disease, FSGS, and lupus nephritis. However, the signaling mechanisms underlying the antiproteinuria effects of DEX have not been well defined. In this study, we further investigated the role of specific signaling mechanisms underlying the protection effects of DEX on podocyte injury. Our results showed that, in podocytes overexpressing TRPC6, cell processes were reduced remarkably. These changes could be rescued by the treatment of the cells with DEX to block TRPC6 channel. Additionally, the podocytes overexpressing TRPC6 treated with DEX showed an improvement in TRPC6 arrangement in the cells and decrease of mRNA expression and protein distribution.

## 2. Materials and Methods

### 2.1. Cells in Culture

Conditionally immortalized mouse podocyte clone (a kindly gift from Professor Peter Mundel, USA, and Professor Jie DING, Peking University First Hospital) was cultured at 33°C in RPMI-1640 containing 10% fetal bovine serum (Gibco, Gaithersburg, MD, USA), 100 U/mL penicillin/streptomycin, and 10 U/mL of mouse recombinant r-interferon (PEPRO Tech, London, UK) and then shifted to 37°C for differentiation by removal of r-interferon [3]. had typical character of mature podocyte after two weeks. In the studies described below, all experiments were performed in growth-restricted podocytes.

### 2.2. Experimental Design

 In order to examine the effect of DEX on PAN-induced podocyte injury, podocytes grown under growth restrictive conditions for 12 days were incubated with media containing 10% FBS in the presence of 1 *μ*mol/L DEX (Sigma Chemical Co.). DEX was not removed until the end of each experiment. To determine if DEX reduced a range of injuries, the following experiments were undertaken after 1 h of DEX incubation: PAN. To determine the mechanisms of podocyte injury induced by PAN, we exposed podocytes to PAN (Sigma Chemical Co) at a concentration of 50 *μ*g/mL. Following a 48 h incubation with PAN in the presence or absence of DEX, then observed and harvested at 8 h, 24 h, and 48 h, respectively. The experiments were all repeated three times.

### 2.3. RT-PCR Analysis

Total RNA was extracted from podocyte using Trizol reagent according to the manufacturer's instruction, and the RNA concentration was determined after the sample was dissolved in diethylpyrocarbonate-treated water. Isolated RNA (1 *μ*g) of each sample was subjected to reverse transcription by using Rever Tra Ace (TOYOBO Co., Japan) according to the manufacture's protocol. The resulting cDNA (3 *μ*L) was used for PCR amplification. The sequence-specific primers were designed and synthesized by Shanghai Invitrogen Biotechnology Co, Ltd. Primers used were as follows. TRPC6 upstream and downstream primers, respectively, were as follows. Forward: 5 GTTAATTGCGATGATCAATAGTT-3. Reverse: 5-GACTTGGTACAAGATTGAAGG-3. Probe: 5-FAM-CCAGGAAATTGAGGATGATGCGGACGTG-BHQ1-3, product length being 143 bp. GAPDH upstream and downstream primers were as follows. Forward: 5-GGTGAAGGTCGGTGTGAACGGAT-3. Reverse: 5-CCACTTTGCCACTGCAAATGGCAG-3. Probe: FAM-CTGGTGACCAGGCGCCCAATACGGCC-BHQ1, product length being 118 bp. The PCR amplification was started with 2 min of denaturation at 94°C, which was followed by 34 cycles (for GAPDH, 30 cycles) of denaturation at 94°C for 30 s, annealing at 55°C for 30 s (GAPDH, 55°C for 30 s), and polymerization at 72°C for 75 s (GAPDH, 30 s). The final extension lasted 7 min at 72°C and then ended at 4°C. PCR products (5 *μ*L) were separated on 1% ethidium bromide-stained agarose gels and later scanned with gel imaging system (Bio Rad Company A). Independent experiment was repeated 3 times.

### 2.4. Western Blot Analysis

Podocytes were lyzed in the buffer containing 1% Tritonx-100, 150 mM NaCl, 1 mM EDTA, 50 mM Tris-HCl (pH 7.7), 1 mM NaF, 1 mM NaVO3, and a protease inhibitor cocktail (Sigma Chemical Co). Seventy-five micrograms of total protein was loaded to run 8% sodium dodecyl sulfate-polyacrylamide gel electrophoresis (SDS-PAGE), and the gel was set up for transfer protein to nitrocellulose membranes (Sigma Chemical Co). Then, the membranes were rinsed in a Tris-buffered saline with 0.02% Tween-20 (TTBS), followed by immersing in 5% low-fat milk. Subsequently, the membranes were incubated with rabbit anti-TRPC6 antibody (Sigma Chemical Co); mouse anti-GAPDH antibody (Sigma Chemical Co). After rinsing three times with TTBS, the membranes were incubated with HRP-conjugated goat anti-rabbit or mouse IgG (Sigma Chemical Co.) for 45 min at room temperature and then developed using ECL chemiluminescence reagent (Sigma Chemical Co.). The specific protein bands were scanned and quantitated using densitometry in relation to the GAPDH, Western Blotting Detection System (GE Healthcare, Chalfont St. Giles, UK). We repeated each Western Blot analysis using protein from three different and separate experiments.

### 2.5. Immunostaining

TRPC6 was fixed with 4% paraformaldehyde, then permeabilized, and blocked with 0.3% TritonX-100 and 5% bovine serum albumin. The primary antibody, rabbit anti-TRPC6 antibody (Sigma Chemical Co), was applied for overnight at 4°C. FITC-conjugated goat anti-rabbit or mouse IgG (Sigma Chemical Co) and the nuclei dye Hoechst were used for 45 min at room temperature. Finally, the coverslips were mounted and images were taken by using a immunofluorescence microscope (Zeiss, Germany). To determine the percentage of the cells in which RPC6 is localized in nuclei, we counted at least 200 nuclei in triplicate in each experiment.

### 2.6. Statistical Analysis

Data were reported as mean ± SD with n equal to the number of experiments. Statistical evaluation was performed using a one-way ANOVA (two-sided test), followed by LSD (equal variances assumed) or Dunnett's T3 (equal variances not assumed) for post hoc test between two groups, and also using the nonparametric tests (Mann-Whitney *U*-test) as a posttest. Values of *P* < 0.05 were considered as statistically significance.

## 3. Results

### 3.1. Effect of DEX on PAN-Induced Podocytes Changes

**Figure 1:**
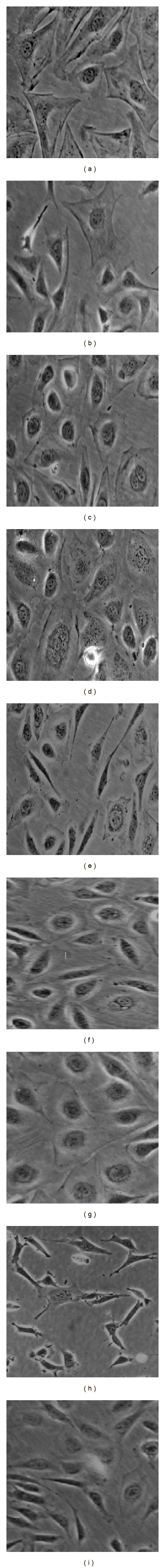
PA-induced podocytes changes at different time points (inverted-phase contrast microscope ×200). Note: (a, d, g): the control group, 8 h, 24 h, 48 h, resp., (foot processes and the connection between podocytes are intact) (b): PAN-induced 8 h; (c): DEX-treated 8 h; (e): PAN-induced 24 h; (f): DEX-treated 24 h; (h): PAN-induced 48 h (foot process retracted and lost, cell interconnected disappeared); (i): DEX-treated 48 h (foot processes and the connection between podocytes are still preserved).

Podocytes were observed and photographed under inverted microscope. The relative area of podocytes was calculated by Image J and SPSS 13.0. The cell bodies and nucleus of PAN-induced podocytes were significantly decreased. The cell bodies, which connected to each other between cells, stretched out like the branches. Foot processes appeared retraction, and the area of PAN-inducted podocytes was significantly reduced to 75% at 8 h (*P* < 0.05); foot processes was obviously retracted, and the cell bodies were reduced to 46% at 24 h (*P* < 0.01); shrinked to 27% (*P* < 0.01), part of foot processes occurred disappearance or lost at 48 h. However, after DEX treated, the area of podocytes was significantly greater at 8 h, 24 h, and 48 h, the difference was significant (*P* < 0.05). Based on these findings, we developed the hypothesis that PAN-induced injury maybe prevented by DEX.  Figure 1.

### 3.2. Effect of DEX on Pan-Induced TRPC6 mRNA Expression

**Figure 2:**
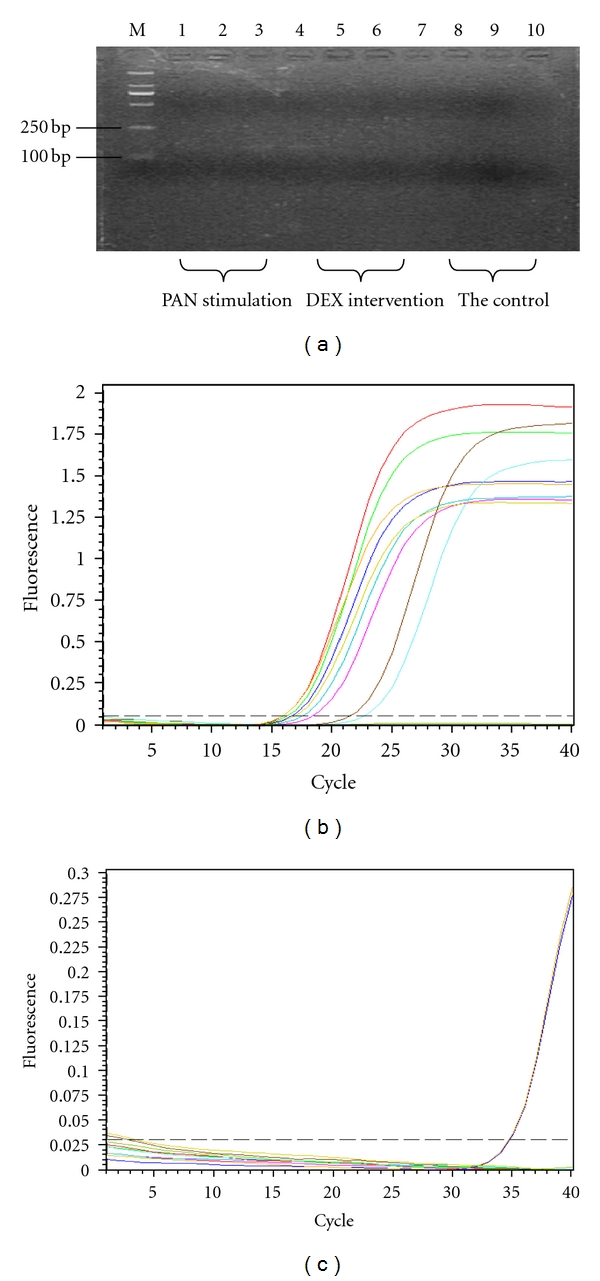
(a) TRPC6 mRNA expression changes at different time points. Note: 1, 4, 7-8 h; 2, 5, 8–24 h; 3, 6, 9–48 h. (b) GAPDH amplification map. (c) TRPC6 amplification map. Note: there was a slight peak in 3rd and 4th samples, ct value reached 34.77, 34.69, indicated that there was Trpc6 mRNA expression in the 3rd and 4th samples, but the expression is low.

TRPC6 mRNA results showed that the target bands can be seen in the 3rd and 4th lanes (150 bp). Our studies showed that there was Trpc6 mRNA expression in the 3rd and 4th samples, but the expression is low, and suggested that TRPC6 mRNA expression is very low under normal circumstances and cannot be shown by the agarose gel electrophoresis. TRPC6 mRNA expression did not change significantly at PAN-induced 8 h and 24 h. But TRPC6 mRNA expression significantly increased at 48 h (*P* < 0.01); TRPC6 mRNA expression did not show significantly changes at DEX-treated 8 h; the TRPC6 mRNA expression was significantly decreased at 24 h and 48 h (*P* < 0.01).  Figure 2(a). 

 Under normal circumstances, TRPC6 expression is very low in podocytes. GAPDH as internal control, Compared with the control group, found that TRPC6 mRNA expression was not significantly changed after PAN-induced 8 h. But TRPC6 mRNA expression slightly increased at 24 h and 48 h (*P* < 0.05); TRPC6 mRNA expression significantly decreased after DEX-treated 8 h, 24 h, and 48 h (*P* < 0.05), Figures 2(b) and 2(c).

### 3.3. Effect of DEX on PA-Induced TRPC6 Protein Expression

**Figure 3:**
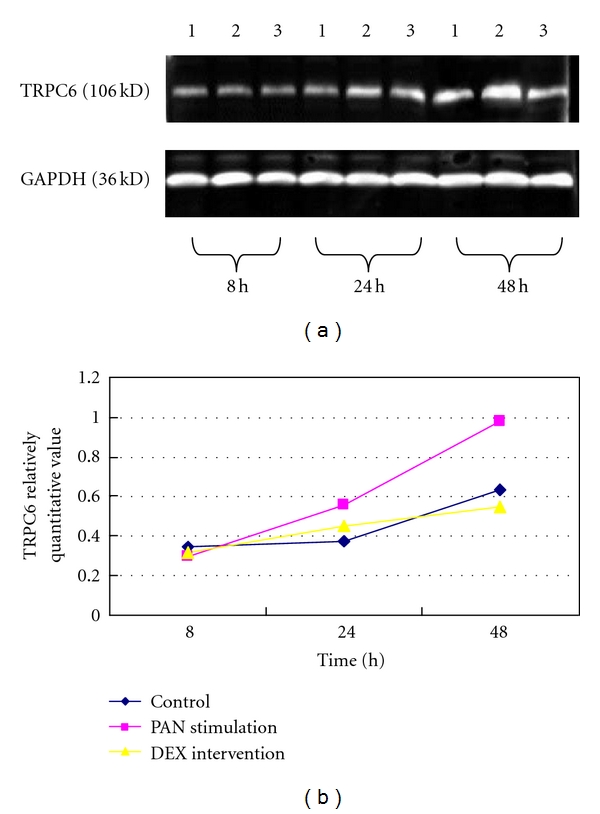
(a) The Western Blot band of TRPC6 and GAPDH at different time points. Note: (1) the control; (2) PAN stimulation; (3) DEX intervention. (b) The Western Blot analysis of TRPC6 and GAPDH at each time point. Note: the Western Blot analysis showed that the protein level of TRPC6 was increased at PAN-induced 24 h and 48 h, the difference was significant, *P* < 0.01; the protein level of TRPC6 was decreased at DEX-treated 24 h and 48 h, the difference was significant, *P* < 0.05.

Western Blot analysis showed that TRPC6 and GAPDH, respectively, have specific band at 36 kd and 106 kd. Under normal circumstances, podocytes have a certain amount of TRPC6 protein expression. Compared with the control, TRPC6 protein expression did not change significantly at PAN-induced 8 h but was higher at 24 h and 48 h (*P* < 0.01); TRPC6 protein expression did not significantly change at DEX-treated 8 h; decreased at 24 h; became normal (*P* < 0.05). The protein expression significantly decreased at 48 h (*P* < 0.01), Figures 3(a) and 3(b). 

### 3.4. Effect of DEX on PAN-Induced TRPC6 Distribution Changes in Podocytes

**Figure 4:**
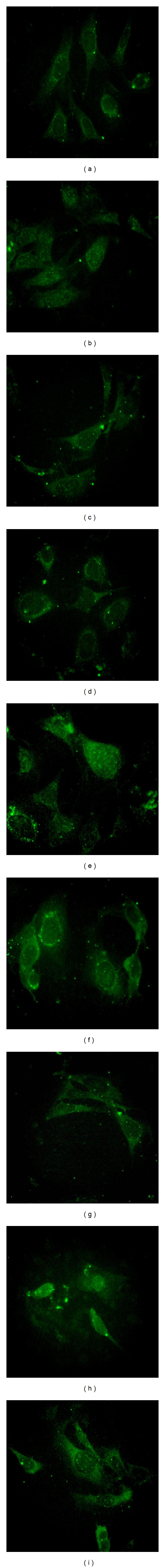
Effects of DEX and PAN on the distribution and protein expression of TRPC6 at different time points (fluorescencemicroscope ×400). Note: (a, d, g) were the control group, TRPC6 was linear and evenly distributed in the podocytes membrane surface, there are some distribution in the cytoplasm; (b) PAN-inducted 8 h; (e) PAN-inducted 24 h, TRPC6 was not continuity distribution along the cell membrane, increased in the cytoplasm; (h) after PAN-inducted 48 h, TRPC6 distribution increased in the cell membrane, part of the cell membrane lost, gathered into a granular, widely distributed in the cytoplasm; (c) PAN-inducted + DEX-treated 8 h; (f) PAN-inducted + DEX-treated 24 h; (i) PAN-inducted + DEX-treated 48 h, TRPC6 was more homogeneous distribution in the membrane at each time point.

TRPC6 was linear and evenly distributed in the control, little in the cytoplasm; at PAN-inducted 24 h, TRPC6 was not continuity distribution along the cell membrane, increased in cytoplasm; at 48 h, TRPC6 distribution increased in cell membrane, part of the cell membrane lost or gathered into granular, widely distributed in cytoplasm. But, after DEX-treated, TRPC6, which significantly improved at different time points, is more homogeneous distribution in the plasma membrane and became normal.  Figure 4.

## 4. Discussion

TRPC6 is a member of the large transient receptor potential superfamily of nonselective cation channels [4, 5]. This superfamily consists of a group of six transmembrane domain-containing ion channels and has been subdivided into six subfamilies; TRPC3; TRPC6, and TRPC7 have about 75% homology, together constitute heteromeric polymer, and form the function channel [6]. Firstly, Winn et al. [7] and Reiser et al. [2], in 2005, determined one of the pathogenic genes of causing familial focal segmental glomerulosclerosis (FSGS). Further studies showed that there was interaction between TRPC6 protein and SD molecules, together constituting the diaphragm hole complex. This finding will make podocyte structural proteins and ion channels linked. Recent studies have also shown that TRPC6 is expressed in foot processes. TRPC6 within the cell body of podocytes and in primary processes in close vicinity to SD was associated with the nephrin, podocin, and CD2AP [8]. TRPC6 is a component of the SD multiprotein complex, involved in the physiologic and pathophysiologic role of podocytes [9].

Glucocorticoids are widely used for the treatment of glomerular diseases characterized by podocyte injury and proteinuria [10]. It has been speculated that glucocorticoids exert therapeutic effects through their immunosuppressive and anti-inflammatory mechanisms [11]. Foot processes and SD contribute to the formation of the glomerular filter. It plays an important role in maintaining the podocyte function. Podocyte injury may result in a leakage of proteins into the urine. So injury to podocytes and their SD typically leads to marked proteinuria. PAN, a podocyte toxin, is wildly used to induce experimental nephrotic syndrome in rats [12–14], In in vitro study, PAN results in foot process effacement and variable dose-dependent apoptosis [15]. As a new SD molecule, TRPC6 is an important molecule for keeping the structure and function of podocytes as well as regulation of signaling transduction in podocytes and interacting with other SD molecules, such as nephrin, podocin, and CD2AP [16–18]. Reiser et al. found that TRPC6 was expressed at the podocyte membrane. In nongenetic forms of glomerular disease, such as minimal change disease (NCD), membranous glomerulonephritis (MGN), and FSGS, TRPC6 overexpresion can directly affect cytoskeletal organization in podocytes [19]. Möller et al. also observed that TRPC6 overexpression correlated with the development of podocyte injury. All of these suggest that TRPC6 overexpression is a pivotal factor resulting in podocyte injury. In the PAN-injected rats, the TRPC6 levels were increased markedly [19]. Knocking-down TRPC6 could effectively prevent the podocytes apoptosis induced by PAN [12]. PAN results in foot process effacement and variable dose-dependent apoptosis [20, 21]. On the basis of these findings, we hypothesized that TRPC6 would also take part in the PAN-induced podocyte injury. DEX resisted podocyte injury via stabilizing TRPC6 expression and distribution. The major purpose of our study was to determine whether DEX mediated the specific cytoprotective effect by blocking TRPC6-signaling pathway and blocking TRPC6 channels maybe beneficial to protect podocytes from injury.

Our study found that, at PAN-induced and DEX-treated 8 h, TRPC6 had no significant changes in protein expression. but was significantly higher at PAN-inducted 24 h and 48 h (*P* < 0.01); TRPC6 protein expression was significantly decreased at DEX-treated 24 h and 48 h (*P* < 0.05). Immunofluorescence staining suggested that TRPC6 showed a linear uniformly distribution in the control group, only a little in the cytoplasm; at PAN-inducted 24 h, TRPC6 was mainly punctate distribution along the membrane and increased in cytoplasmic. At 48 h, TRPC6 distribution increased in the cell membrane, part lost, gathered into a granular, widely distributed in the cytoplasm; TRPC6 was more homogeneous distribution after DEX treatment. The distribution was significantly weaker in cytoplasm. The difference, which is compared with other podocyte molecules, was that TRPC6 expression strengthened in PAN-inducted podocytes, the distribution increased but reduced after DEX-treated. This is the special position of TRPC6 as a cation channel protein. But the specific mechanism is not very clear, pending further study. Our results suggested that TRPC6 might be a pharmacological target of maintaining the function of podocytes in the future. So blocking TRPC6 channels may be a new therapeutic strategy in proteinuric renal diseases.

 Our evidence also suggests that blocking TRPC6 channels may be of therapeutic benefit in proteinuria. This creates the exciting possibility that blocking TRPC6 channels within the podocyte may translate into long-lasting clinical benefits in patients with FSGS. Our study suggested that TRPC6 filled the damage signal, which was induced by PAN, and then activated the downstream molecules, caused the injury signal transduction, so that foot process responds to retracted and lost. TRPC6 abnormal expression and distribution led to imbalance of TRPC6 channels and function. The changes of podocyte filtration rate caused the occurrence of proteinuria; the DEX may have made the downstream molecules, which have been activated, inactivated, podocytes restored their original structure by blocking TRPC6 signal channels, thus maintained SD structural and functional integrity by stabilizing TRPC6 expression, and played a protective role in anti-proteinuria.

In summary, TRPC6 overexpression may be one of the fundamental changes in podocytes leading to proteinuria and impairment of renal function. Perhaps, blocking the TRPC6 channel may be candidate for diagnosis and therapeutic target of renal diseases. Our study suggested that TRPC6 signal pathway participated in the signal transduction mechanisms of DEX inhibiting podocyte injury induced by PAN. DEX stabilized TRPC6 expression through binding to its receptor. Our study will provide more clearly theoretical basis for the molecular mechanism of DEX antiproteinuria and also provide new insights into the special beneficial effects of DEX on podocyte injury.
